# The application of Interleukin-2 family cytokines in tumor immunotherapy research

**DOI:** 10.3389/fimmu.2023.1090311

**Published:** 2023-03-02

**Authors:** Yangyihua Zhou, Guiqi Quan, Yujun Liu, Ning Shi, Yahui Wu, Ran Zhang, Xiang Gao, Longlong Luo

**Affiliations:** ^1^ Department of Medical Laboratory, School of Medicine, Hunan Normal University, Changsha, Hunan, China; ^2^ State Key Laboratory of Toxicology and Medical Countermeasures, Beijing Institute of Pharmacology and Toxicology, Beijing, China; ^3^ Cancer Research Institute, Department of Neurosurgery, School of Basic Medical Science, Xiangya Hospital, Central South University, Changsha, China

**Keywords:** interleukin, IL-2 family, tumor immunotherapy, engineered cytokines, clinical development

## Abstract

The Interleukin-2 Family contains six kinds of cytokines, namely IL-2, IL-15, IL-4, IL-7, IL-9, and IL-21, all of which share a common γ chain. Many cytokines of the IL-2 family have been reported to be a driving force in immune cells activation. Therefore, researchers have tried various methods to study the anti-tumor effect of cytokines for a long time. However, due to the short half-life, poor stability, easy to lead to inflammatory storms and narrow safety treatment window of cytokines, this field has been tepid. In recent years, with the rapid development of protein engineering technology, some engineered cytokines have a significant effect in tumor immunotherapy, showing an irresistible trend of development. In this review, we will discuss the current researches of the IL-2 family and mainly focus on the application and achievements of engineered cytokines in tumor immunotherapy.

## Introduction

**Figure 1:**
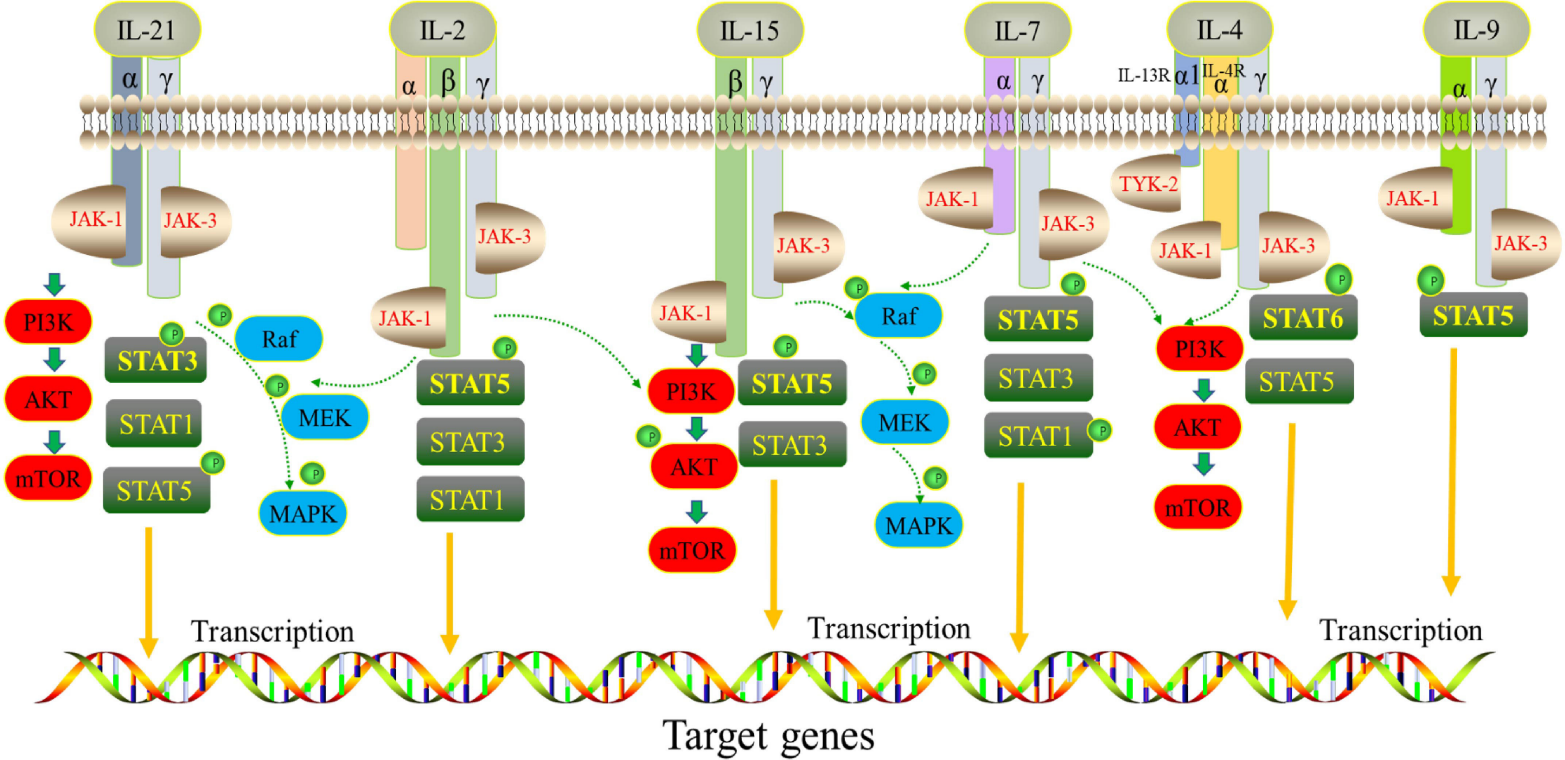
Signaling network of the IL-2 cytokine family. Receptors of IL-2, IL-15, IL-21, IL-4, IL-7 and IL-9 share a common γ chain subunit. They phosphorylate various STAT proteins downstream by activating JAK/STAT signaling pathway.

Interleukin (IL)-2 is a member of the cytokine family. In common with other receptors in the family, namely IL-4, IL-7, IL-9, IL-15 and IL-21, IL-2 shares a common receptor γ chain (i.e., IL-2Rγ). IL-2 family cytokines are pleiotropic, type I four α-helix-bundle cytokines secreted by hematopoietic cells and stromal cells (1, 2). They are indispensable for the functioning of innate immunity, adaptive immunity, and some actions beyond immune systems, especially playing vital functions in the development of T, B, and natural killer (NK) cells. Typically, the γ chain, as the common receptor of the IL-2 family, forms heterodimers or heterotrimers with other subunits, for example IL-2Rα/β, IL-15Rα, IL-21Rα, IL-4Rα, IL-7Rα, IL-9Rα (3). Moreover, γ chains can transmit signals through the Janus kinase (JAK)-signal transducer and activator of transcription (STAT) pathways (2). IL-2, IL-15, IL-21, IL-7 and IL-9 can induce the phosphorylation and activation of STAT1, STAT3 and STAT5 proteins. However, IL-21 induces a higher level of pSTAT3 expression, while the other cytokines activate mostly STAT5. IL-4 leads to the activation of both STAT5 and STAT6 (4). The IL-2 family cytokines also regulate specific genes involved in the phosphoinositide 3-kinase (PI3K)-AKT-mammalian target of rapamycin (mTOR) pathway and in mitogen-activated protein kinase (MAPK) signaling (5) **Figure 1**.

A deficiency of tumor infiltrating lymphocytes (TIL) in the tumor microenvironment is an important obstacle for those patients with tumors resistant to cancer immunotherapy. Several studies on IL-2 family cytokines have elucidated their biological functions and anti-tumor effects. In particular, IL-2 plays a large role in activating anti-tumor immune responses in the tumor microenvironment (6, 7). Thus, IL-2 family cytokines profoundly affect the survival, proliferation, differentiation and function of T lymphocyte subsets, including CD8^+^, CD4^+^, and NK-T cells, which play central roles in anti-tumor responses (8, 9). Dating back to the 1990s, IL-2 was approved as the first cancer immunotherapy drug for metastatic renal cell carcinoma and metastatic melanoma. To date, IL-2 and interferon-α (IFN-α) are the only two cytokine drugs for the treatment of cancers approved by the U.S. Food and Drug Administration (FDA). Yet, more IL-2 family cytokines, such as IL-15, IL-21, IL-7 have been reported to play a synergistic and unique role in mediating the activation, proliferation and differentiation of T and NK cells during immune response. For example, IL-21 strongly drives proliferation and expression of the effector molecules of NK cells (10). However, in practical applications, use of the IL-2 family cytokines faces three difficulties. First, cytokines typically have a small molecular weight and short half-life that can limit drug distribution and metabolic rate. Second, cytokines are immune agonists and lead to systemic side effects. Third, cytokines used individually as a single drug typically fail to target tumors and thus cannot effectively activate the anti-tumor immune response in the tumor microenvironment. Accordingly, extensive efforts have been focused on producing engineered proteins with desired beneficial properties to address the limitations of these natural molecules (11). Most notably, two exciting directions in cytokine drug development include improving its binding affinity using designated receptors and regulating its bias in recognition with key signaling molecules. This article reviews the latter type of engineering strategy. In addition, some researchers have tried to conjugate polyethylene glycol (PEGylation) to cytokines to prolong its *in vivo* half-life. Some researchers have also constructed antibody-cytokine fusion proteins to achieve half-life extension and allow tumor targeting (8, 12, 13).

In this article, we provide an overview of the structural composition and functional characteristics of each member of the IL-2 family of cytokines, briefly discussing their potential value in tumor immunotherapy. We then summarize the diversity, promising prospects and the stage of clinical application of engineered IL-2 family cytokine-based immunotherapies. Of note, the structure of this article is not sorted by IL-2 family number size but is instead presented based on the number of studies for that family member, from most to least. The aim of this study is to review the molecular characteristics of the IL-2 family of cytokines, the research progress in tumor immunotherapy and the current status of clinical research, with the goal of providing a comprehensive and broad vision to guide researchers or drug research institutes in future studies and to promote the clinical application of IL-2 family cytokine drugs in tumor immunotherapy.

## IL-2

IL-2 was first discovered in the supernatant of activated T cells in 1976 and initially named T-cell growth factor (14). Its receptor contains three different chains, the IL-2 receptor α chain (IL-2Rα, CD25), the IL-2 receptor β chain (IL-2Rβ, CD122) and the IL-2 receptor γ chain (IL-2Rγ, CD132). These receptors have different affinities with IL-2. IL-2Rα is expressed on activated lymphocytes and binds to IL-2 with low affinity. IL-2Rβ and IL-2Rγ combine to form the IL-2Rβ/γ complex, typically on the surface of memory T cells and NK cells, and this complex binds to IL-2 with medium affinity. When the three receptors IL-2R α/β/γ are co-expressed on activated T cells and regulatory T (Treg) cells, IL-2 binds to them with high affinity to form a 4-membered complex. Analysis of the three-dimensional structure of this quaternary complex revealed that IL-2 is initially associated with IL-2Rα and then recruits IL-2Rβ and IL-2Rγ. The medium affinity and high affinity receptor forms can transmit IL-2 signals and perform corresponding functions (15). The γ-chain is encoded by the IL-2RG gene, and mutations in this gene cause X-linked severe combined immunodeficiency disease (XSCID), which is characterized by decreased or absent T cells, NK cells and non-functional B cells (16, 17). Surprisingly, T and NK cell numbers are normal in IL-2 deficient patients and IL-2 knock out mice (18, 19). This led researchers to hypothesize that there must be cytokines sharing the IL-2Rγ, which was later confirmed and includes IL-4, IL-7, IL-9, IL-12, and IL-21 (20). Given this, these six cytokines are placed into the common cytokine receptor γ-chain family, also known as the IL-2 family of cytokines.

The function of IL-2 has been widely investigated. IL-2 is required for the generation of Treg cells, namely the CD4^+^Foxp3^+^CD25^+^CD127^low^ population in the thymus, and is required for Foxp3^+^ T cells to exert survival and suppressive functions in autoimmune diseases (21), such as chronic graft-versus-host disease (cGVHD), type 1 diabetes (T1D) and systemic lupus erythematosus (SLE). The primary role of IL-2 is to induce immune responses by stimulating the proliferation and differentiation of effector T cells, memory T cells and NK cells (22). Since the emergence of biotechnology, IL-2, as a biological agent for the treatment of cancer, has laid the foundation for the use of other recombinant cytokines to treat tumors. High-dose IL-2 is crucial to expand cytotoxic lymphocytes but its therapeutic value is limited by the need for frequent dosing, which can lead to harmful side effects such as hematologic and hepatic toxicity, mental confusion, hypoxia and respiratory distress (23, 24). Therefore, a variety of methods have been developed to prolong the half-life and weaken the limitations of the treatment window, while harnessing the immunostimulatory effects and overcoming unfavorable Treg toxicity of IL-2 for patients suffering from malignant tumors. A summary of IL-2-based therapies used for cancer treatment follows.

In the 1990s, IL-2 (Aldesleukin) was approved by the FDA for the treatment of metastatic kidney cancer (1994) and melanoma (1998). Aldesleukin has a certain anti-cancer effect but has not been widely used because of its toxic side effects. To address potential clinical side effects, much research has focused on engineered IL-2 cytokines. Some engineered IL-2 are mutated to silence the binding activity to CD25, only exposing the IL-2-binding sites of CD122 and CD132 to selectively stimulate CD8^+^ memory T cells and NK cells. Those types of cells only express high levels of IL-2Rβγ dimer with intermediate affinity (25).

Alternatively, for the treatment of autoimmune diseases, investigators sought to expand Treg cells that efficiently express IL-2Rαβγ by masking the binding sites of IL-2 to CD122 and CD132, only exposing the binding sites to CD25 (26). Solomon et al. designed an optimized antibody, anti-CD25^NIB^ that selectively depletes Treg cells without affecting IL-2-STAT5 signaling and can thus enhance effector activation and antitumor immunity (27). Arenas-Ramirez and colleagues designed a monoclonal antibody against human IL-2, called NARA1 (28), and formed a NARA1-IL-2 complex. This antibody occupies the CD25 epitope of IL-2, blocking the interaction between IL-2 and CD25 and effectively stimulating CD8^+^ memory T cells and NK cells. More interestingly, to address the issue of IL-2 clinical therapeutic mutations, Silva and colleagues constructed a completely new functional protein structure similar to IL-2/IL-15, called Neo2/15, by *de novo* design. The protein contains 100 amino acid residues and has a completely different topology and sequence from human and mouse IL-2, which means it can only bind and activate IL-2Rβ and IL-2Rγ but cannot bind and activate IL-2Rα; this binding property has enhanced its therapeutic properties and reduced its side effects. Compared with native IL-2 and engineered Super-2 (29) (also called IL-2 superkine; it ablates the interaction between CD25 and IL-2 by mutation), Neo2/15 is better able to activate human and mouse primary T cells. A single dose of Neo-2/15 showed dose-dependent delayed tumor growth in melanoma and colon cancer mouse models (30).

At present, several engineered IL-2 molecules or antibodies are in the clinical research stage. Currently, two phase I clinical trials are ongoing to evaluate the safety and toxicity of RO7296682 (a Treg cell depleting antibody) as a single agent (NCT04158583) and in combination with Atezolizumab for participants with advanced solid tumors (NCT04642365). Moreover, the engineered fusion protein ALKS 4230, consisting of a circular arrangement of IL-2 and IL-2Rα extracellular domains, has been designed to target and activate effector cells expressing medium affinity for IL-2Rβγ. ALKS 4230 does not bind to IL-2Rα on the cell-surface, unlike recombinant interleukin (rIL)-2, and thus has a milder activating effect on immune cells. In both preclinical mouse models and in *in vitro* experiments with primary human cells from healthy people and advanced cancer patients, ALKS 4230 induced greater expansion and activation of NK cells and memory-phenotype CD8^+^T cells and decreased levels of Treg cells and proinflammatory cytokines, such as IL-6 and tumor necrosis factor (TNF). ALKS 4230 exerted better anti-tumor efficacy relative to rIL-2 or a vehicle control in a B16F10 melanoma implanted lung model (31). In addition, in a pharmacokinetic and pharmacodynamic study in cynomolgus monkeys, administration of ALKS 4230 resulted in superior expansion of immune-protective cells, including CD56^+^ NK cells, terminal effector CD8^+^ cells and effector memory cells (32). These data provide further support of the clinical evaluations of ALKS 4230. For the moment, ALKS 4230 as a monotherapy or a combination with immune checkpoint inhibitors are being tested in clinical trials (**Table 1**).

**Table 1:** Generalization of the clinical trials of engineered IL-2 cytokines.

Drug	Cytokine	NCT Number	Phase	Immunotherapy Combinations	Conditions
**ALKS 4230** **(Nemvaleukin Alfa)**	IL-2	NCT04592653 NCT03861793 NCT02799095 NCT04144517 NCT04830124 NCT05092360	1,2,3	Anti-PD-1 Antibody (Pembrolizumab)	Advanced Solid Tumors
**THOR-707** **(SAR 444245)**	IL-2	NCT04009681 NCT05104567 NCT05179603 NCT04913220 NCT05061420 NCT04914897	1,2	Anti-EGFR antibody Cetuximab Checkpoint inhibitor Pembrolizumab (Keytruda®) Carboplatin	Metastatic solid tumors Gastrointestinal cancer B cell lymphoma Advanced skin cancers Lung cancer
**NKTR-214** **(Bempegaldesleukin)**	IL-2	NCT04936841 NCT02983045 NCT03785925 NCT03635983 NCT04955262 NCT03745807 NCT04730349 NCT04969861 NCT03835533 NCT03729245 NCT02983045 NCT04646044 NCT03138889 NCT04209114 NCT04540705 NCT04052204	1,2,3	Anti-PD-1 (Nivolumab Pembrolizumab) Anti-CTLA-4 (Ipilimumab) Tyrosine Kinase Inhibitor (Abozantinib) Chemotherapy	Advanced Solid tumors High-grade Glioma Metastatic Head and Neck Cancer Coronavirus Disease 2019
**L-19-IL-2**	IL-2	NCT05329792 NCT02076633 NCT02086721 NCT02076646 NCT01198522 NCT01253096 NCT02938299 NCT01058538 NCT04362722 NCT01055522 NCT03567889	1,2,3	Gemcitabine Dacarbazine L19TNF	Basal Cell Carcinoma Squamous Cell Carcinoma Malignant Melanoma Solid tumors
**RO6895882**	IL-2	NCT02004106 NCT02350673	1	Atezolizumab	Neoplasms Solid Tumors
**RO7284755**	IL-2	NCT04303858	1	Anti-PD-1 Atezolizumab	Advanced and/or Metastatic Solid Tumors
**FAPIL2v (Simlukafusp alfa、RO6874281)**	IL-2	NCT03386721 NCT03875079 NCT02627274 NCT03063762	1,2	Anti-PD-L1 Anti-PD-1 Trastuzumab Cetuximab Atezolizumab Bevacizumab	Solid Tumor Breast Cancer Renal Cell Cancer of Head and Neck
**hu14.18-IL2**	IL-2	NCT03209869 NCT00590824 NCT03958383 NCT00109863 NCT00003750 NCT00082758 NCT01334515	1,2	Anti-PD-1 (Nivolumab Anti-CTLA-4 (Ipilimumab) Radiation Therapy Isotretinoin	Neuroblastoma Osteosarcoma Melanoma Unspecified Childhood Solid Tumor

**Figure 2:**
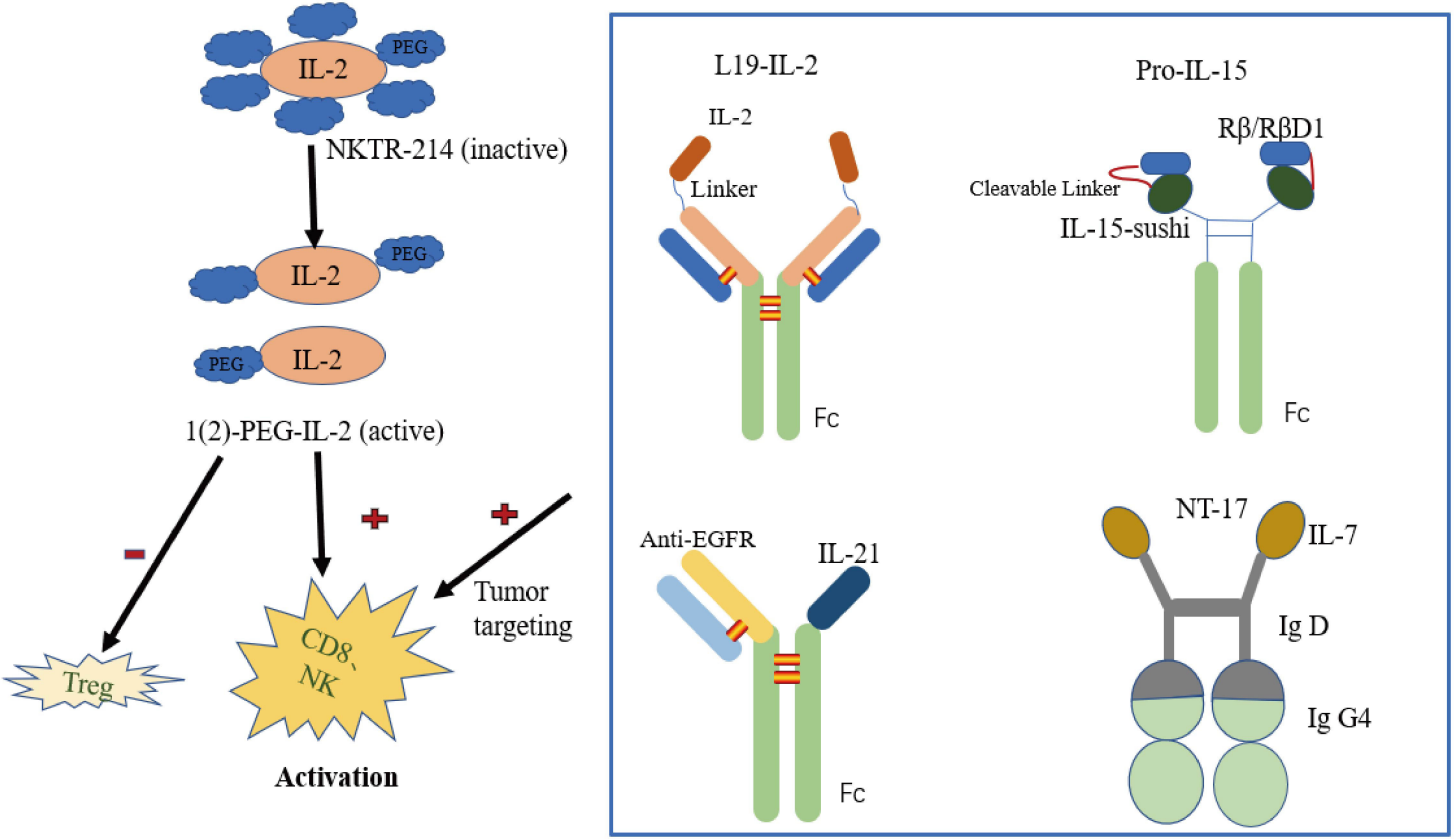
Representative engineering cytokine patterns.

In addition to changing the binding properties of IL-2 receptors by mutation or computer-aided design, PEG modification is another main direction in IL-2 drug development. NKTR-214 (bempegaldesleukin) is a new generation of pegylated IL-2 drugs (33) (**Figure 2**). It innovatively links six PEG molecules with IL-2 to form a prodrug known as NKTR-214, which has no drug activity of its own. In the human body, the PEG group is irreversibly shed to release the active components of 2-PEG and 1-PEG. Both have a high affinity for IL-2Rβγ receptors and low affinity for the IL-2Rαβγ receptors, thus reducing the promotion of Tregs proliferation while stimulating the immune system (activating CD8^+^T cells and NK cells) (34). At the same time, pegylated modification prolongs the drug half-life. This induced upregulation of the expression of programmed cell death protein 1 (PD-1) molecules on CD8^+^ tumor-reactive T cells in the first human study. Nonetheless, an objective response rate (ORR) of zero was observed in 28 recruited patients with advanced metastatic renal cell carcinoma and melanoma treated with NKTR-214 (35). In contrast, patients in another study treated with standard IL-2 had positive ORR values of 57.14% ORR for patients with metastatic melanoma and 100% ORR for patients with metastatic kidney cancer (36). Since NKTR-214 alone has little effect, it has been speculated that NKTR-214 and immune checkpoint inhibitors (anti-PD-1, anti-CTLA-4) have a synergistic effect in resisting tumor, and this has been strongly supported in some preclinical (37, 38) and clinical experiments (**Table 1**).

Another IL-2 drug candidate, THOR-707 (SAR 444245), is a “non-α” recombinant protein of IL-2 variants that, through specific modifications, allows polyethylene glycol chains (PEGs) to adhere to specific sites, preventing it from binding to immune receptors IL-2R-α (CD25). This specifically elicits large-scale accumulation of infiltrating CD8^+^ lymphocytes and NK cells in tumor tissues, leading to a dose independent reduction in tumor growth in C57BL/6 mice bearing B16-F10 tumors (39). Thus, THOR-707, as a single drug or combined with immune checkpoint inhibitors (CPI), is also being developed for use with a variety of solid tumors (**Table 1**).

Antibody-cytokine fusion proteins are a new class of biologics that target cytokines to tumor cell sites, increasing leukocyte infiltration in the tumor microenvironment (40). The first antibody-cytokine fusion proteins were reported as early as the 1990s, fusing IgG antibodies with multiple interleukins, tumor necrosis factors, and interferons (41–43). The L19-IL-2 fusion protein delivers IL-2 to fibronectin targeted by L19 monoclonal antibodies (**Figure 2**). Fibronectin is expressed in the extracellular matrix of vascular endothelial in most solid tumors and malignant blood diseases, so the infusion of L19-IL-2 improves the cytokine index within tumor tissue and increases the median survival in mouse models of sleeve cell lymphoma (44). L19-IL-2 has also been proposed, and is being evaluated, clinically as a single agent or in combination with other tumor-targeted monoclonal antibodies in many solid tumors (**Table 1**). Slightly different from what was reported above, Klein and colleagues designed an IL-2 mutant (IL-2v) that does not bind to CD25 (45) but fuses with the antibody CH11A 1-2F1, targeting the carcinoembryonic antigen (CEA) to form the antibody-cytokine fusion protein CEA-IL-2v (RG7813). This immune protein preferentially binds to CEA rather than IL-2R, making it highly accumulated at the tumor site, preferentially activating CD8^+^ T cells and NK cells. Moreover, CEA-IL-2v (RO6895882) as a drug in combination with Atezolizumab has been applied to CEA-positive solid tumor Phase I clinical trials (**Table 1**).

The PD-1-IL-2v fusion protein (RO7284755), a PD-1 targeted IL-2 variant immunocytokine developed by the company Hoffmann-La Roche, can deliver IL-2 mutants to PD-1^+^ T cells. PD-1-IL-2v treatment leads to greater expansion of proliferative and cytotoxic effector cells compared to non-PD-1-targeted IL-2v and anti-PD-1. Clinically, a phase IA/IB study is in progress to estimate the safety and anti-tumor activity of RO7284755 both alone and in combination with Atezolizumab in patients with advanced and/or metastatic solid tumors (**Table 1**). Another novel immunocytokine designed by Hoffmann-La Roche is simlukafusp alfa (FAP-IL-2v). Here, an IL-2v mutein is fused with the high-affinity fibroblast activation protein α (FAP) human IgG1 antibody 4B9, allowing this fusion protein to deliver the IL-2 variant to the surface of cancer-associated fibroblasts and pericytes that highly express FAP, without preferential activation of Treg cells. In murine models, FAP-IL-2v accumulates in tumors and activates CD8^+^ T cells, NKp46 NK cells, and CD68-positive macrophages. It is effective at controlling tumor growth and prolonging the median survival of tumor-bearing mice when used in combination with other therapeutic antibodies, such as anti-mPD-L1, anti-mCD40 and antibody-dependent cellular toxicity (ADCC)-competent antibodies (46). Currently, there are four clinical trials related to this drug (**Table 1**).

Hu14.18-IL-2 is an immunocytokine (IC) fusion protein consisting of a humanized anti-disialoganglioside (GD2) monoclonal antibody and human recombinant IL-2 (hrIL-2) developed to exert superior anti-tumor activity against melanoma and neuroblastoma. As expected, targeted IL-2 enhanced the ADCC of hu14.18 mAb (anti-GD2) to efficiently inhibit tumors in murine models (47, 48). A phase II trial of the hu14.8-IL-2 fusion protein for the treatment of refractory or recurrent neuroblastoma disease had a 21.7% complete response rate in 23 patients, which is promising. Other related clinical trial results are given in **Table 1**.

In summary, we have made a brief overview of some representative engineered IL-2 cytokines that are expected to be candidates for the next generation of immunotherapies. In reality, there are also an increasing number of experiments focusing on IL-2 cytokine in combination with adaptive cell transfer (ACT), cancer vaccines (49), chemotherapeutic drugs and surgical operation. Those studies will not be covered in detail in this review.

## IL-15

IL-15 was first reported as an important cytokine of the IL-2 family in the 1990s, when it was found in the supernatant of two cell lines, CV-1/EBNA and HTLV-1-associated HuT-102. IL-15 has a molecular size of 14-15 kD and can stimulate T cell proliferation and induce NK cell activation (50). It is widely expressed by various cells, including myeloid cells, fibroblasts and epithelial cells but, surprisingly, not by T cells (51). The IL-15 mature protein is a 114 amino acid peptide, with 97% and 73% homology to the IL-15 sequences of monkeys and mice, respectively (52). IL-15 has similar biological properties to IL-2 because they share the same receptor signaling components. IL-15 receptors consist of a specific IL-15Rα, a transmembrane protein and a common intermediate affinity IL-15Rβγ (also known as IL-2Rβγ) receptor. Soluble IL-15 can bind to IL-15Rα alone with high affinity (10^-11^ M) and is found on the cell surface in the form of IL-15/IL-15Rα complexes. In fact, IL-15Rα serves as a repository for IL-15 by recycling the free IL-15 protein and making it available for *cis*- or *trans*- expression (53). IL-15 has a unique mechanism of action. It typically interacts with its receptors in *cis-* and *trans-* ways. For *cis*-presentation, IL-15 binds to the receptors co-expressed on T cells, NK cells, natural killer T (NKT) cells and B cells; this is necessary for survival and proliferation of CD8^+^ memory phenotype T cells and antigen-specific memory CD8^+^ T cells, among others (54). For *trans-*presentation, IL-15Rα from one subset of cells (such as dendritic cells) present IL-15 to adjacent NK and CD8^+^ T cells expressing only IL-15Rβγ. Compelling evidence suggests this is the main mechanism by which IL-15 generates biological activities *in vivo*. Such binding mediates functional effects similar to those of IL-2, including stimulating proliferation of CD8^+^ T cells, NK cells, cytotoxic lymphocytes and induction of immunoglobulin synthesis by B cells through the JAK-STAT pathway (JAK 1 and JAK 3, STAT3 and STAT5) (55). IL-15 can also activate the MAPK pathway and the PI3K/Akt/mTOR pathway. Since IL-15 does not bind to IL-2Rα (CD25), it does not lead to the proliferation and activation of Treg cells, but it does stimulate the expansion of NK and effector T cells, and especially supports the survival of CD8^+^ memory T cells (56). Unlike the unique role of IL-2 in activation-induced cell death (AICD), a major mechanism underlying peripheral self-tolerance, IL-15 has a competing role in inhibiting IL-2-mediated T cell death that contributes to enhanced immune memory (57). Moreover, IL-15 can protect neutrophils from apoptosis, promoting the maturation of dendritic cells (58, 59). In all, IL-15 can promote cellular expansion, enhance the functions of effector and cytotoxic immune cells, and promote the secretion of effector molecules, such as IFN-γ, TNF-α, XCL1, granzyme and perforin (60). This suggests that IL-15 is an attractive candidate for cancer immunotherapy.

Soluble IL-15 (sIL-15, monomer IL-15) is secreted at very low levels with a half-life of less than 40 minutes (61). In addition, high-dose injection of this single agent can cause toxicities. Since IL-15 appears in the form of a heterodimer in the circulation after binding with the soluble IL-15Rα (62), various attempts have been made to use the concept of natural *trans-*presentation of IL-15 to ameliorate the biological activity of IL-15. For example, when soluble IL-15 is injected simultaneously with the fusion protein sIL-15Rα-Fc (formed by the IL-15Rα and IgG1 Fc regions), its ability to activate proliferation of mouse memory phenotype CD8^+^ T cells is 50 times greater than that of IL-15 alone (63). The construction of a complex of sIL-15/IL-15Rα co-expressed on engineered cells by gene therapy significantly extends the half-life of sIL-15. Much greater bioactivity is displayed by sIL-15/IL-15Rα because it creates a conformational change that potentiates IL-15 recognition by the βγ receptors on T cells. The sIL-15/IL-15Rα complex can thus rapidly induce strong and selective amplification of CD44^high^ memory CD8^+^ cells and NK cells (64). Furthermore, fusion proteins (RLI and ILR), in which IL-15 and IL-15R alpha-sushi domain (Ile 31 to Val 115) are attached by a flexible linker, are even more potent than the combination of IL-15 with sIL-15R alpha-sushi (65). In many experimental murine models, the fusion protein used alone or in combination with checkpoint inhibitors, such as anti-PD-1 Abs, delays tumor growth, can induce tumor regression and prolongs survival in mice (66).

A recombinant chimeric protein consisting of IL-15, IL15Rα sushi domain, and the apolipoprotein A-I (Sush-IL15-Apo) has also been developed. The scavenger receptor type I class B receptor (SR-B1) expressed on the surface of tumor cells is a receptor for ApoA-I and this allows SR-B1 to aggregate chimeric proteins on the surface of tumor cells to deliver IL-15 to NK and CD8^+^ T cells. Furthermore, simultaneous infusion of this chimeric protein with anti-EGFR antibodies can increase the ADCC effects of monoclonal antibodies and reduce the number of colon cancer tumor cells in the abdominal cavity (67). Many more examples of the combined use of IL-15 and other cytokines in tumor treatment exist. IL-15 and IL-21 may synergistically stimulate NK-92 cells, release more immune cell-derived exosomes, and mediate stronger cell killing activity of NK cells (68). IL-15 and IL-2 can upregulate the expression of the NKG2D activating receptor on immune lymphocytes and augment NKG2D-relevant NK cells anti-tumor cytotoxicity in melanoma patients (69). Recombinant human(rh)IL-15, with rhIL-12 and rhIL-18 may prime blood NK cells to differentiate into memory-like NK cells and potentiate their responses against cancer (70).

Another next generation drug of IL-15 in preclinical trials is tumor-conditional IL-15 pro-cytokine (Pro-IL-15), designed by the team of Professor Fu Yangxin of Tsinghua University (**Figure 2**). Pro-IL-15 consists of an extracellular domain of IL-15Rβ fused to the N-terminus of sIL-15-Fc *via* a matrix metalloproteinase (MMP) cleavable linker. This keeps Pro-IL-15 inactive until it accumulates in the tumor and is cleaved by the high MMP environment of the tumor microenvironment. This pro-cytokine reduces on-target off-tumor toxicity and induces greater regression of MC-38-bearing tumors in murine models (71). Recently, Lu et al. (72) constructed a new immunocytokine (LH01) by fusing the IL-15 receptor alpha-sushi domain/IL-15 (IL-15N72D) complex with the antibody against programmed death-ligand 1 (PD-L1). LH01 not only had a prolonged half-life and improved the tumor-targeting distribution of IL-15, it also overcame resistance to PD-L1 blockade and reduced both CT-26 and MC-38 tumor burden in mouse models. This may be attributed to LH01 increasing the activation of cells involved in innate immunity and adaptive immunity.

More projects are modifying the IL-15 molecule to overcome its limitations (*in vivo* short half-life, no *trans*-presentation by IL-15Rα) in clinical applications. For example, the IL-15 super-agonist, N-803 (formerly ALT-803), developed by ImmunityBio assembles an IL-15 mutant protein (IL15N72D), an activating mutation of IL-15Rα sushi domain, and IgG1-Fc together to allow *trans-*presentation and decreased renal clearance. IL15N72D induces stronger phosphorylation of the JAK1 and STAT5 proteins and anti-apoptotic activity, resulting in a four to five times increase in biological activity over natural IL-15 (73). N-803 effectively boosts CD34^+^ hematopoietic progenitor cell (HPC)-NK cell proliferation in a dose-dependent manner, induces IFN-γ production, and augments tumor cell death in mice bearing human ovarian cancer cells (74). In addition, N-803 treatment increases the expression of PD-L1 in immune cells *in vivo*, so N-803 combined with anti-PD-L1 is well-tolerated by intraperitoneal infusion. Due to the increase in the number of immune cells in the lungs and spleen, the MC38-CEA tumor load is reduced, the lung metastasis of 4T1 triple negative breast cancer is significantly inhibited, and the survival period is prolonged (75). We have found 28 studies for N-803 in clinical trials and made display in **Table 2**.

**Table 2:** Summary of clinical trials involving IL-15 and IL-7.

Drug	Cytokine	NCT Number	Phase	Immunotherapy Combinations	Conditions
**N-803** **(IL-15-Superagonist, IL15N72D, ALT-803)**	IL-15	NCT04808908 NCT04340596 NCT04505501 NCT02989844 NCT04385849 NCT03022825 NCT03853317 NCT04247282 NCT02138734 NCT03493945 NCT03520686 NCT04927884 NCT04491955 NCT05096663 NCT04898543 NCT03563157 NCT03563170 NCT03228667 NCT04290546 NCT03387085	1,2,3	Anti-PD-1 (Pembrolizumab, Nivolumab) TriAd vaccine Bacille Calmette-Guérin vaccine NHS-IL12 Chemotherapeutic drugs	HIV Infections, COVID-19 Acute Myelogenous Leukemia (AML) Bladder Cancer, Head and Neck Neoplasms Colorectal Cancers Solid Tumors
**BJ-001**	IL-15	NCT04294576	1	PD-1 or PD-L1 inhibitor	Locally Advanced/Metastatic Solid Tumors
**NKTR-255**	IL-15	NCT04616196 NCT04136756 NCT05327530	1,2	Rituximab, Avelumab Daratumumab, Sacituzumab Govitecan Cetuximab, M6223	Non-Hodgkin Lymphoma Multiple Myeloma Head and Neck Squamous Cell Carcinoma Colorectal Cancer Locally Advanced or Metastatic Urothelial Carcinoma
**NT-I7/GX-I7** **(Efineptakin alfa)**	IL-7	NCT05075603 NCT04332653 NCT04588038 NCT04594811 NCT03901573 NCT05191784 NCT04810637 NCT04730427 NCT04588038 NCT04893018 NCT04501796 NCT04984811	1,2	Anti-PD-1 (Pembrolizumab, Nivolumab) Anti-PD-L1 (Atezolizumab) Anti-VEGF (Bevacizumab)	Advanced Solid Tumors Refractory Diffuse Large B-cell Lymphoma Triple Negative Breast Cancer Non Small Cell Lung Cancer Recurrent Head and Neck Squamous Cell Carcinoma Recurrent Glioblastoma HIV Infection, COVID-19

BJ Bioscience, Inc. has developed the first tumor targeting IL-15 fusion protein, BJ-001, with a global patent. BJ-001 is a fusion protein of IL-15 and RGD (Arg-Gly-Asp-D-Phe-Lys, an integrin receptor antagonist polypeptide), which enables it to be enriched in tumors with high expressions of αvβ3, αvβ5 and αvβ6 integrin. Pharmacodynamic studies show that BJ-001 has obvious anti-tumor effects in the subcutaneous transplantation tumor model of hucct1 human cholangiocarcinoma cells in BALB/C nude mice. Currently, a phase I study is ongoing to assess the safety and tolerability of BJ-001, as a single agent and in combination with PD-1 or PD-L1 inhibitor in adult patients with locally advanced or metastatic solid tumors (**Table 2**). Another IL-15 candidate drug is NKTR-255, a conjugate of a novel polyethylene glycol-recombinant human IL-15 (rhIL-15). Compared to rhIL-15, NKTR-255 induced a 2.5- and 2.0-fold increase in NK and CD8^+^ T cells, respectively, in multiple cancer models. NKTR-255 can also utilize IL-15Rα for *cis*- presentation on CD8^+^ T cells acting as an IL-15Rβ agonist and potentially advancing immunotherapies for cancer treatment (76–78). The first phase I human clinical trial is currently underway to examine the efficacy of NKTR-255 as monotherapy or in combination with daratumumab or rituximab for hematologic malignancies. The study includes 46 patients and will evaluate the safety, toxicity and maximum tolerated dose (MTD) in the dose-escalation phase that minimizes side effects. Another 72 patients will be enrolled to explore the recommended Phase II dose (RP2D) (79) (**Table 2**). At the same time, approved combinations of rIL-15 with other agents in the clinic, such as the combination of monoclonal antibody alemtuzumab and rhIL-15, can further improve the therapeutic efficacy of adult T-cell leukemia (ATL) (NCT02689453). A phase I study combining IL-15 expressing autologous NKT cells and GD2 specific CAR (chimeric antigen receptor) is being evaluated for its efficacy in treating children with neuroblastoma (NCT03294954).

In conclusion, IL-15-based agents are potent cancer immunotherapies, especially with the emergence of next-generation IL-15 cytokines. However, we need more attempts to obtain IL-15 engineered drugs with better clinical therapeutic value.

## IL-21

IL-21, a multifunctional type I cytokine belonging to the IL-2 family, was discovered in 2000. The IL-21-coding gene is localized on the long arm of human chromosome 4 (4q26-q27). After transcription and translation, the precursor molecule containing 162 amino acids is formed; the mature functionally active molecules with 131 amino acids of 14 kD are formed once cleaving occurs at position Gly 31. IL-21 is produced by CD4^+^ T cell subsets, such as Th17, T-follicular helper cells (Tfh), NKT cells, and by CD8^+^ T cells in some viral infections. IL-21R is a heterodimeric complex composed of a specific IL-21Rα chain and the common shared γ-chain (CD132) of the IL-2 family. As the unique receptor of IL-21, the IL-21Rα encoding gene encodes 583 amino acid residues that form a transmembrane glycoprotein of 75 kD. The structure of the IL-21Rα-chain is similar to that of the IL-2Rβ-chain and IL-4Rα-chain (80, 81). IL-21R is located in normal lymphoid tissues such as the thymus, lymph nodes, and splenocytes. Interestingly, expression of IL-21R has also been detected in other non-lymphoid tissues, such as bone marrow cells, thyroid cells (82) and keratinocytes. Both IL-21Rα-chain and γ-chain are important signal subunits of IL-21. The IL-21-IL-21R signaling pathway induces an increase in phosphorylation of the Janus family tyrosine kinases JAK1 and JAK3, and further activates downstream STAT3, STAT1 and, to a lesser extent, STAT5 proteins. The dimerization of phosphorylated STAT proteins are transferred to the nucleus and modulate the expression of IL-21 related genes, such as cMyc, Bax (STAT3-dependent proapoptotic genes), Bcl-2 and Bcl-XL (two anti-apoptotic genes) (83), and INFG (repressor of cytokine signaling 3 and 1 (SOCS3 and SOCS1) that negatively regulates the JAK-STAT pathway) (84). STAT3 and STAT1 synergistically activate IL-21 downstream gene expression, but in some cases this phenomenon is reversed. For example, IL-21-mediated activation of STAT1 can inhibit STAT3-dependent IL-21 expression (84). Simultaneously, IL-21 can also activate two different pathways, PI3K-AKT and MAPK, to regulate the differentiation and polarization of immune cells and related non-immune cells.

Generally speaking, IL-21 exerts pleiotropic functions on various immune cells. On the one hand, IL-21 can induce Th17 and mediate the development of autoimmune diseases (85). On the other hand, IL-21 can induce Tfh cell differentiation, and Tfh can induce germinal center formation, long-lived plasma cell (LLPC) production (86), and eventually induce memory B cells to secrete high-affinity antibodies (87). In addition, IL-21 may promote the maturation of memory CD8^+^ T cells, and then sustain and enhance the anti-tumor cytotoxicity of effector CD8^+^ cells and NK cells. This is closely related to the IL-21 signaling pathway downstream transcription factor basic leucine zipper ATF-like transcription factor (BATF) (88, 89). Moreover, IL-21 promotes the conversion of macrophages from the M2 phenotype to the M1 phenotype. Unlike IL-2, IL-21 does not expand Treg cells *via* the suppression of Foxp3 expression (90). Thus, the same dose of IL-21 in clinical practice has lower lethal toxicity and systemic side effects compared to other members of the IL-2 cytokine family (IL-2, IL-15). Nevertheless, IL-21 reduces the expression of MHC class II molecules in dendritic cells (DCs), further inhibiting the activation and maturation of DCs at the early phase of the immune response (91).

IL-21 has dual roles in tumor therapy. Although IL-21 enhances the development of colitis-associated colon cancer and leads to inflammation in head and neck squamous cell carcinoma (92, 93), several clinical phase I/II studies (94–96) and preclinical results continue to indicate that IL-21 can stabilize disease and induce stronger anti-tumor activity in many cancers, including B-cell Hodgkin’s lymphoma, melanoma, renal cancer, metastatic colorectal cancer, ovarian cancer and non-small cell lung cancer (97). Moreover, a recent preclinical study suggests that IL-21 can potentiate the ability of granulocyte-macrophage colony-stimulating factor (GM-CSF) to stimulate anti-tumor immunity in mice to resist bladder cancer (98). IL-21 is required for the generation of tumor-infiltrating CD8^+^ T cells expressing CX3CR1 characterized by cytolytic function and enhanced secretion of effector molecules such as interferon-γ and granzyme B (99). IL-21 may enhance NK cell-mediated antibody-dependent cell-mediated cytotoxicity more than IL-2 and IL-15 (100). Overall, preclinical studies have begun focusing on the development of IL-21 as an adjuvant drug for tumor treatment. Infusion of high-dose (200 µg) IL-21 does not lead to the production of inflammatory cytokines or endothelial tissue damage in mice, and only generates minimal vascular leakage. However, an equivalent dose of IL-2 leads to vascular leakage in mice as manifested by the infiltration of inflammatory cells and increase of cytokines, such as TNF-α, IL-6 and IFN-γ in serum (101). Although IL-21 has a wider safety dose window, it has the following problems as an antineoplastic agent. First, recombinant human IL-21(rhIL-21) has a short half-life; reports suggest half-life is only 2 hours in the human body. Second, rhIL-21 has a systemic profile and a high affinity for IL-21R, making it difficult to achieve effective enrichment at the tumor site; thus, safety and efficacy are not well balanced. Lastly, rhIL-21 has a certain immune activation effect in theory and as its single-agent anti-tumor effect is limited, a risk of inducing autoimmune diseases exists.

To counterbalance the high affinity binding of IL-21 to non-tumor tissues expressing IL-21R, researchers have tried fusing IL-21 with tumor-targeting or T cell-targeting antibodies to form fusion proteins. This improves anti-tumor efficiency by decreasing off-target cytotoxicity and extends the survival of clinically advanced cancer patients. Several examples exist in the literature. In a recent study, IL-21 fused with anti-EGFR or anti-CD20 exerted a more effective anti-tumor effect (102, 103) (**Figure 2**). Tumor-targeting antibodies can effectively deliver drugs to the tumor location, and cytokines have certain chemotaxis and activation functions that can more effectively activate the anti-tumor activity of the immune system and improve the tumor microenvironment immune infiltration. Consider αCD20-IL-21 as an example. This drug format not only prolongs the serum half-life of IL-21, but also effectively overcomes single-drug resistance, promotes NK-cell activation and cytotoxic function facilitating IFN-γ secretion and inducing stronger apoptosis of lymphoma cells. The investigators also reported on an antibody-cytokine fusion protein drug, PD-1Ab21, that combines anti-PD-1 antibody with IL-21 in a bifunctional drug format. It is precisely because this form of medicine can more effectively direct IL-21 to tumor-specific T cells and promote the formation and proliferation of memory stem cell-like T cells with CD44^low^-CD62L^high^ phenotype that the therapeutic effect is greatly improved and the side effects reduced (104).

Combining an engineered design of a highly attenuated IL-21 mutein variant (R9E:R76A) and a PD-1 blocking antibody prolongs the half-life and allows a longer duration between clinical treatment cycles (105). IL-21-αHSA, an engineered immunocytokine in which a nanobody targeting human serum albumin (HSA) is fused to the carboxyl terminal of recombinant human IL-21 (rhIL-21), has half-life extended and stability increased compared to rIL-21. Whether used as a monotherapy or in combination with immune checkpoint blockades (PD-1, T cell immunoglobulin, or ITIM domain (immunoreceptor tyrosine-based inhibitory motif)), enhanced anti-tumor efficacy results (106). In addition, IL-21 stimulates NK cells and leads NK cells to have stronger ADCC effects on cetuximab-treated pancreatic cancer cells (107). Phase I/II clinical trials include the IL-21/PD-L1 fusion protein AMG-256 (NCT04362748) initiated by Amgen, BMS-982470 (rIL-21) in combination with BMS-936558 (anti-PD-1) for solid tumors (NCT01629758) and rIL-21 combined with rituximab or sorafenib for non-Hodgkin’s lymphoma (NCT00347971) or metastatic renal cell carcinoma (NCT00389285). Shanghai Junshi Biopharmaceutical recently extended the half-life of IL-21 by fusing an anti-HAS single-domain antibody to improve the distribution of the drug in the tumor microenvironment. This drug, JS401 (rhIL-21-HAS single-domain antibody fusion protein), has been approved for clinical trials. Lastly, a patient presenting with stage III melanoma refractory to the monotherapy of adoptive transfer of CD8^+^ cytotoxic T lymphocytes (CTLs) and anti-CTLA-4 achieved a complete remission (CR) and remains disease-free 5 years later after infusion with IL-21-primed polyclonal CTL plus CTLA-4 blockade (108). Lamentably, so far, none of these next-generation candidate IL-21 drugs have entered the clinical stages.

To summarize, IL-21, the most recently discovered of the IL-2 family cytokines, has great anti-tumor potential and has attracted considerable attention from the biomedical community. Yet, the clinical development of IL-21 lags compared with other cytokines in the IL-2 family, such as IL-2 and IL-15. Hence, finding a way to ensure that IL-21 can achieve higher and more effective therapeutic effects under the premise of using the maximum biosafety dose requires more effort. Collectively, IL-21 as a monotherapy and in combination or fusion with other vaccines, cytokines or antibodies can increase the function of NK and CD8^+^ T-cells in the tumor, suggesting drug development based on IL-21 has a bright future.

## IL-4

IL-4, also known as a pleiotropic type 2 cytokine (the others include IL-5 and IL-13), was first discovered in 1982 as a 4-α helix bundle pleiotrophin secreted by CD4^+^ T cells, Th2 cells, basophils, eosinophils and mast cells (myeloid cells) (109). hIL-4 cDNA encodes 153 amino acid residues and yields a 129 aa-secreted protein, with a molecular weight of 12-20 KDa depending on the degree of N-terminal glycosylation (110). The human IL-4 and mouse IL-4 gene are located on chromosome 5 and chromosome 11, respectively. The related receptor IL-4R has three different forms. The type I IL-4R consists of an IL-4Rα chain and a common IL-2Rγ chain and is expressed in endothelial cells, epithelial cells, and liver and brain tissue; it binds exclusively to IL-4. The type II IL-4R is expressed only on non-hematopoietic tissues is composed of an IL-4Rα chain and an IL-13Rα1 chain that can simultaneously bind to IL-4 and IL-13, respectively (111–113). Type III IL-4R includes the above three types of receptors (IL-4Rα, IL-13Rα1, IL-2Rγ). IL-4Rα is a cytokine-binding receptor for IL-4. Once IL-4 and IL-4Rα combine with high affinity, the IL-4/IL-4Rα complex then binds to the second receptor, either IL-2Rγ or IL-13Rα1 to form a functional receptor complex (114).

IL-4 binding to receptors can also activate multiple signaling pathways, such as the JAK-STAT, insulin receptor substrate (IRS)2/PI3K/Akt/mTOR signaling pathways. It is worth noting that the STAT6 protein of a downstream signaling pathway is recruited and phosphorylated to mediate the majority of IL-4 functions (115). Ligand (IL-4 or IL-13) binding to IL-4Rα and IL-13Rα1 correlate with the differentiation of naïve T cells into Th2 effector cells [the major contributor to B-cell help and IgE antibody production (116)] and eosinophil cells, which are highly related to some autoimmune diseases, including asthma (117) and multiple sclerosis patients (118). IL-4 also boosts survival and proliferation of mast cells and polarizes macrophages to M2 macrophages. These functions all suggest that IL-4 plays a significant role in propagating its pathogenesis in the occurrence and development of allergies or tumors (119). Therefore, many anti-IL-4 drugs are designed to treat related diseases by inhibiting the IL-4-IL-4R signal pathway.

In cancer, however, IL-4 was originally used as an immunotherapeutic drug against malignant tumors. IL-4 elicited a potent antitumor response in plasmacytoma, mammary adenocarcinoma and Hodgkin lymphoma in murine models (120, 121). IL-4 and CpG oligonucleotide therapy enhanced expression of MHC and co-stimulatory molecules in DCs, promoting the production of IFN-γ and suppressing the outgrowth of established melanoma tumor cells (122). Moreover, a variant of IL-4, Super-4, developed for higher affinity binding to the γ chain, has increased potency and activation of T cells and B cells. This kind of IL-4 superkine was aimed to be used as a super agonist to treat cancer (123). However, in recent years, an increasing number of clinical data suggest that IL-4 primarily plays a tumor-promoting effect in most tumors (115, 124–127).

Overall, the pleiotropy and complexity of IL-4 limits its clinical development as a candidate drug for tumor therapy. On the contrary, many attempts are being made to treat various allergic diseases or/and autoimmune diseases by reducing the level of IL-4 and blocking the binding of IL-4 to its receptor.

## IL-7

IL-7 differs from other members of the IL-2 cytokine family in that it is mostly produced by non-haematopoietic stromal cells instead of leukocytes, although small amounts are produced by DCs. IL-7, with a molecular weight of 25 kD of fractional soluble globular proteins is abundantly expressed in thymic cells. The human IL-7 gene encodes a protein of 177 amino acids and shares 55% homology with the murine IL-7 gene, which encodes a 154 amino acid protein of 18 kD (128). IL-7R is an iso-dimer complex consisting of an IL-7Rα (CD127) chain and the common IL-2Rγ chain (129–131). IL-7Rα belongs to the hematopoietin receptor family and is widely expressed throughout the lymphatic system, except in mature B cells and in developing T and B cells (132). IL-7Rα can bind to IL-7 and thymic matrix-derived lymphopoietin (TSLP) (133). The γ chain is expressed on all hematopoietic cell types and integration of IL-2Rγ and IL-7 is necessary for the signaling and functioning of IL-7 (134). The IL-7-IL-7R signaling pathways include JAK/STAT, PI3K/Akt and MAPK pathways (135).

IL-7 has pleiotropic functions and elicits different regulatory actions depending on its localization and adjacent microenvironment. In this review, we focus on characteristics related to the use of IL-7 as a therapeutic drug in the context of tumors. As an anti-tumor therapy, IL-7 can further the survival, growth and differentiation of T cells, maintain internal environmental stability and boost the development of memory precursors (7). In viral infections, IL-7 promotes the production of memory-type T cells, and in the presence of IL-15 and the blocking of IL-2, IL-7 can accelerate the proliferation of T cells expressing the CD44 memory phenotype (136). In addition, IL-7 stimulation increases cytolytic and noncytolytic activity and cytokine production of CD8^+^ T cells purified from patients with hepatocellular carcinoma, by suppressing the expression of PD-1 (137). IL-7 can also prevent apoptosis *in vitro* with an increase in Bcl-2, an anti-apoptotic protein (138).

Clinically, a completed phase I trial supported by National Institutes of Health Clinical Center used recombinant IL-7 (CYT 99 007) to treat patients with refractory solid tumors since IL-7 may stimulate white blood cells to kill tumors (NCT00062049). In a phase II study, recombinant glycosylated human IL-7 (CYT107) combined with vaccine therapy treatment stopped tumor growth in patients with castration-resistant prostate cancer (NCT01881867). Another ongoing study is assessing the best dose of recombinant IL-7 to promote T, NK, and B cell recovery in patients after transplantation of cord blood (NCT03941769). A randomized phase II clinical trial to observe the regeneration of lymphocytes and various T cell populations in lymphopenic sepsis patients intravenously injected with human recombinant glycosylated IL-7 (CYT107) is documented (NCT03821038); however, this clinical trial was terminated in October of last year.

The research and development situation of IL-7 as the next-generation anti-tumor drugs in recent years is not optimistic. The exception is one experiment that found the co-expression of IL-7 and CCL19, a chemoattractant for T cells and DCs, in CAR-Tcells can extend cell survival and enhance the anti-tumor efficacy of CAR-T cells in mice (139). Simultaneously, a clinical follow-up study of CD19 CAR-T cells expressing IL-7 and CCL19 for relapsed or refractory B cell lymphoma in humans has been launched (NCT04833504). Subsequently, a phase I clinical trial combining this therapy with PD-1mAb was conducted in patients with relapsed or refractory diffuse large B cell lymphoma (DLBL) (NCT04381741). The long-term remission rate of DLBL was greatly improved. In addition, efineptakin alfa (NT-17/GX-17), the only next-generation drug of IL-7 developed by NeoImmune Tech (**Figure 2**), and the world’s first and only long-acting recombinant human IL-7 (rhIL-7), can augment cytotoxic CD8^+^ T cells expansion, increase IFN-γ production, decrease the number of T_reg_ cells and prolong the survival of C57BL/6 mice glioma models (140). Moreover, more than ten clinical trials are studying the safety, tolerability, and anti-tumor activity of NT-17 as a single drug or in combination with immune checkpoint inhibitors and other monoclonal antibodies in patients with glioblastoma, B-cell lymphoma, breast cancer, non-small cell lung cancer and the recent Covid19 (**Table 2**).

Finally, IL-7 involves a variety of diseases, including autoimmune diseases, infections and cancers (141, 142). It plays different roles in different tumors, either promoting or inhibiting the occurrence and development of cancer cells (135). As a result, it is difficult to modify IL-7 to design the next generation of engineered IL-7 cytokines against tumor growth. We still have a long way to go to understand the immune protective effects of IL-7 in various tumors and the optimal dose in clinical cancer patients without side effects.

## IL-9

In 1988, a “T cell growth factor” secreted by T helper cells (Th9) was discovered and called P40 glycoprotein, now called IL-9 (143). IL-9 is a 14 kD glycoprotein with 144 amino acids. The human IL-9 gene is located on chromosome 5, while the mouse IL-9 gene is in a syntenic region on chromosome 13 (144). IL-9 can be produced by activated naïve CD4^+^ T cells, mast cells, group 2 innate lymphoid cells (ILC2s), Treg cells, T follicular helper cells and TH17 cells, among others (145–150). IL-9R consists of a unique IL-9Rα chain and the common IL-2Rγ chain (151). IL-9 needs to bind to these two receptor subunits at the same time to mediate further effects. The downstream signaling pathways include the phosphorylation of JAK, STAT1, STAT3, and STAT5 transcription factors, all related to the multiple biological functions of IL-9. Two other pathways, MAPK and PI3K-AKT, have been reported but are not well studied (152). IL-9 is reported as a multifunctional cytokine. Previous studies have shown that IL-9 has the ability to stimulate cell proliferation, so it is not surprising that IL-9 plays certain roles in allergic inflammation, tumorigenesis and the development of cancers such as lung, breast, thyroid, and colon cancer (153, 154). Specifically, IL-9 can promote cancer cell proliferation and protect tumor cells from apoptosis. But recently, evidence increasingly suggests that IL-9 also exerts anti-tumor effects. For example, Th9 cells produce IL-9 in solid tumors and enhance tumor-specific cytotoxic T lymphocyte (CTL) responses *in vivo* (155). Another study found that IL-9 inhibits subcutaneous colon cancer and prolongs survival time in BALB/C mice by recruiting tumor infiltrating lymphocytes (such as tumor-specific CD8^+^ T cells and CD8^+^ granzyme B^+^ cells) to the tumor microenvironment, where they regulate T-cell function and kill tumor cells (156). In addition, IL-9 can suppress tumor development; injection of recombinant IL-9 into wild-type mice bearing melanoma or lung carcinoma leads to reduced tumor mass (157). Further, in IL-9 receptor-deficient murine models, B-16 melanoma grows faster. Moreover, Fang et al. (158) found that IL-9 could promote HTB-72 or SK-Mel-5 cell apoptosis by upregulating expression of the anti-proliferation molecule p21 and the pro-apoptosis molecule TNF-related apoptosis-inducing ligand (TRAIL) in human melanoma, thus controlling melanoma growth. IL-9 can also act directly on tumor cells expressing IL-9R, such as squamous cancer (SqC) cells and cervical cancer, to directly kill these tumor cells (159).

Overall, although IL-9 plays a beneficial role in some cancers (such as melanoma) by inducing not only innate immunity but also adaptive immunity, its tumor-promoting and inflammatory effects limit its clinical development. To date, no clinical trial has been approved to analyze the anti-tumor efficacy of IL-9 in patients with cancer. Greater exploration and research into IL-9 is clearly needed.

## Future perspective

Although engineering cytokines have made breakthroughs in recent years, such as improving the tumor targeting or reducing the systemic toxicity of systemic drug delivery, as far as I know, the application of engineering cytokines in tumor treatment still faces the following problems. Firstly, different types of tumors (such as breast cancer, gastric cancer, and pancreatic cancer) have different shapes, densities, margins, relationships with surrounding tissues, and malignancies. Therefore, it is urgent to study the molecular mechanisms of different types of tumor microenvironments and their sensitivity to drug therapy. And design targeted drug delivery strategies or develop new drug delivery technology platforms to exert the maximum anti-tumor effect of drugs. Secondly, considering that tumor cells have strong heterogeneity and relatively complex tumor microenvironment, the application of those engineered drugs will still face the risk of drug resistance, and more combined therapies (such as immune checkpoint blockade, oncolytic viral therapy, adoptive T cell therapy, and tumor vaccines) need to be developed and tested urgently. Lastly, different types of cytokines have different activation effects on immune cells. For example, IL-2 cytokines tend to activate CD8^+^ T cells proliferation to produce effector function, while IL-21 cytokines better activate cells with central memory phenotype, which are more persistent and have higher anti-tumor activity *in vivo*. Therefore, in the process of engineering cytokine design and clinical application, the activation bias of cytokines on immune cells should be fully considered to determine the specific application scenarios and precautions of different engineering cytokines. Moreover, it is more noteworthy that the undisclosed mechanism behind the different functional responses of IL-2, IL-15, IL-21and IL-7 needed to be further explored.

## Conclusion

In recent years, cancer immunotherapy has become standard therapy in clinical setting, benefitting more cancer patients. Cytokine-based immunotherapy is preferred among the immunotherapy strategies and has become an important area of drug research and development. In particular, members of the IL-2 cytokine family have received considerable attention since the 1990s. IL-2, IL-15, and IL-21 in the IL-2 cytokine family play a positive role in anti-tumor effects, and IL-4, IL-7 and IL-9 possess pleiotropic functions, with diverse roles in cancer immunity as they can have pro-tumorigenic functions as well as anti-tumorigenic characteristics. The surveillance and clearance of malignant cells are closely related to the dynamic balance of various cytokines in the IL-2 cytokine family. The early application of these natural cytokines faced numerous challenges, such as a short half-life, fast clearance rate and narrow safe dose window (with high-dose infusion causing systematic toxicity). Fortunately, the development of next-generation of cytokine therapeutic drugs has improved the physiological defects of these cytokines. The strategy of combining cytokines with other therapies, such as CAR-T cells, vaccines and immune checkpoint inhibitors, have proven promising in their ability to completely eliminate tumors.

Current FDA-approved cytokine therapeutics only include IL-2 and IFN-α (160), which have been shown to have significant objective survival remission rates in patients with metastatic renal cancer and melanoma. The remaining cytokines were terminated when were used as single drugs or combined with adoptive T-cell immunotherapy, vaccines, and other therapeutic agents because no obvious clinical effects were observed in phase I and II clinical trials. Thus, in future, we need to make more efforts to address the common and individual problems, such as the drug-making properties of cytokines, improving the half-life of cytokine drugs, tumor targeting, and, especially, activating anti-tumor immunity while effectively balancing the safety of drugs *in vivo*. Related forms of drug development models include, but are not limited to, biased IL-2 family protein drugs, low-affinity cytokine drugs, and antibody-cytokine fusion proteins. These forms of drug are expected to solve some of the current challenges with cytokine drug development, but it is not enough. We are certain more innovative cytokine drug design and development will bring about greater changes. We look forward to this.

## Author contributions

Conceptualization: XG, RZ, LL; Writing-original draft: YZ; Writing – review & editing: GQ, YL; Validation and Visualization: NS, YW. All authors contributed to the article and approved the submitted version.
